# Drafting of Physicians under the Selective Service Act

**Published:** 1918-02

**Authors:** Robert M. Codd

**Affiliations:** Buffalo


### Drafting of Physicians Under the Selective Service Act.

By ROBERT M. CODD, JR., B. S. M. E., L.L. B.

The sovereign power or duly constituted authority of any land may in time of internal stress or foreign conflict call to its support the property, services or even the lives of its subjects.

Under the feudal system we find serfs attached to the land and the land and all of its attachments the absolute property of a feudal lord, who in turn owes his unquestioned allegiance to his sovereign.

Where this supreme power has been resumed by the people they, by that act, become masters of their property and conduct. But man is gregarious, and our present day interdependence would be impossible, had we any such individual freedom or even that of the patriarchates or family control.

When this individual freedom of action is voluntarily surrendered to promote the public good, a sovereign power is created, given certain duties and obviously the authority to enforce them, restraining individual action and thought, and even taking private property for the public welfare. The measure of this authority may be found in a Magna Charta, a Bill of Rights or a Constitution. But where it is once delegated it is all powerful, and when it speaks all must acquiesce.

For example, we find a captain of a ship at sea, in time of storm throwing overboard a part of the cargo to save the balance, which on the arrival of the vessel at its destination will not be delivered to the consignee, but will be subjected to the law of general average, or be sold in bulk and the proceeds divided pro rata among the various owners of the entire cargo. So, too, we find a fire marshal razing a building to stay the progress of a fire which has become a danger to the entire community.

What holds for property is equally good for personal services and even the lives of a people. So in the case Johnson vs. Dodd, in 56 New York Reports, page 81, we find it stated "The government has the right to require the personal services of its citizens for the public defense. Minors and adults and even those apprenticed are alike subject to this supreme authority."

The law martial and the court martial now succeed the codes of Civil Procedure and the usual courts for the enforcement thereof.

In the spring of 1917 Congress spoke. War was declared. Under the Public Welfare clause, so-called, and under the implied authority that the right to declare war carried with it the right to raise armies, a National Army was provided for by the terms of the Selective Service Act. By the terms of this act, certain exemptions from compulsory military service were permitted, as employees of the Federal, State and other government officers, and others. The entire body of men, called to the colors are regarded as a part of the National Army, must report as such, when required to do so, or suffer stigma and punishment as deserters, they being subject to the law martial. If they are of one of the exempt classes they may show this by affidavits or otherwise, and the various local boards will allow the exemption.

What of the doctor? This and other occupations call for a special training, then an examination as to proficiency and a state license to practice. Should the exemption board view all applications for relief from the draft alike? The man who has devoted time and money to acquire his profession, and has received a state permit to follow it up, should he be sent to the front irrespective of his potential ability for special usefulness in his community.?

The exemption boards are given semi-judicial powers. There is some question as to this delegation of the powers of courts, either civil or martial, to these boards. But it is obvious that a physician should and would receive exemption at their hands, should he hold a fatal number in the government's great lottery. This exemption on account of his calling, in a measure conscripts him to a form of public service.

Not so the man trained and who has taken a medical degree, but never followed it up by its practice. His value in any special line is extremely problematic. No exemption board could with justice excuse him from service were he drawn for such.

The act calling into being the National Army is termed Selective. Obviously the men taken are selected with reference to their duties and relations to the communities from which they come. Thus a doctor with a considerable practice would not be called, while a less fortunate and junior member of the profession might have an opportunity to gain valuable experience and be of the greatest service to his fellows by being called to the colors. Here his professional ability would be recognized and he would be set to work along the line of his abilities, for were he thus trained the military authorities would never permit him to practice with a bayonet when he could do so with a lancet.

